# Emerging roles of MiR-133a in human cancers

**DOI:** 10.7150/jca.48769

**Published:** 2021-01-01

**Authors:** Yu-Ting Hua, Wen-Xiu Xu, Hui Li, Min Xia

**Affiliations:** 1 Department of Gastroenterology, Wuxi People's Hospital Affiliated to Nanjing Medical University, 299 Qingyang Road, Wuxi, Jiangsu 214023, China.; 2 Department of General Surgery, the First Affiliated Hospital of Nanjing Medical University, Nanjing 210029, P.R. China.

**Keywords:** cancer, miRNA, MiR-133a

## Abstract

MicroRNAs (miRNAs) can post-transcriptionally regulate the expression of cancer-relevant genes via binding to the 3'-untranslated region (3'-UTR) of the target mRNAs. MiR-133a, as a miRNA, participate in tumorigenesis, progression, autophagy and drug-resistance in various malignancies. Based on the recent insights, we discuss the functions of miR-133a in physiological and pathological processes and its potential effects on cancer diagnosis, prognosis and therapy.

## Introduction

Cancer is a worldwide health concern due to its high mortality. Treatments of cancer, especially that in advanced stage, lay a tremendous economic burden for both patients and the society 1.Therefore, it is urgent to explore novel, highly specific biomarkers and targeted treatments for cancer. MiRNAs, a class of noncoding RNA (ncRNA) about 22-nucleotide RNAs in size, are highly conserved molecules regulating gene expression post-transcriptionally by binding to the 3'-untranslated region (3'-UTR) of target message RNAs (mRNAs) 2 and participating in tumorigenesis, proliferation, invasion and drug resistance in cancer 3. Among the miRNAs, miR-133a has been considered as a tumor suppressor and a biomarker for prognosis of various cancers, such as osteosarcoma 4, esophageal cancer (EC) 5, colorectal cancer (CRC) 6, non-small cell lung cancer (NSCLC) 7, bladder cancer 8, breast cancer 9 and gastric cancer (GC) 10.

The miR-133 family (miR-133a, miR-133b), also classified as myomiRNAs for its role in skeletal and cardiac muscle development, is located on the 18th (miR-133a-1), 20th (miR-133a-2), and 6th (miR-133b) chromosomes, and transcribed as bicistronic transcripts with miR-1-2, miR-1-1, or miR-206. MiR-133 is involved in a variety of diseases, including cardiac hypertrophy, heart failure, cardiac arrhythmia, muscular dystrophy and cancers 11, 12.

## The biochemical and molecular properties of MiR-133a

MiR-133a was first experimentally characterized in mice and found highly conserved in mice, flies and human. MiR-133a-1 and miR-133a-2 possess identical nucleotide sequences, while miR-133b differs from miR-133a by a single nucleotide at the 3' end (GU→A) 13.

## The methods for the detection of MiR-133a

It is well known that the ways of detecting microRNAs mainly include high throughput sequencing, quantitative real time polymerase chain reaction (RT-qPCR) and microarrays^14^. RT-qPCR technology is the most frequently applied to detect the expression of miR-133a in cancer tissues and cell lines. As a circulating microRNA, miR-133a can also be deteced in body fluid, like blood and gastric juice^15^, suggesting its value as a non-invasive biomarker.

## The interactions of MiR-133a with other molecules

MiR-133a plays the tumor suppressive role via tageting and regulating the genes like SOX4, EGFR, FSCN1, COL1A1 and so on (**Table 1**). Accumulating evidence demonstrated that long non-coding RNAs (lncRNAs) could function as endogenous miRNA sponges or competing endogenous RNA by binding to miRNAs and regulating their function. We summarized the lncRNA-miR-133a interactions in **Table 2.** Whether circular RNA (circRNA) is involved in miR-133a-mediated tumor inhibition is worth studying.

## MiR-133a in cell proliferation and apoptosis

Malignant tumors are manifested as activated cell proliferation and reduced cell apoptosis. Low-frequent ultrasound-targeted microbubble destruction (UTMD) of miR-133a injected in mice with breast cancer significantly suppressed tumor growth 9. MiR-133a could negatively regulate the cell cycle and further suppress cell proliferation in breast cancer via targeting EGFR. The over-expression of miR-133a in MCF-7 and MDA-MB-231 cells could decrease G2/S phase and interfere DNA synthesis through the EGFR/Akt signaling pathway 16. MiRNA-133a induced NSCLC cell apoptosis and decreased cell proliferation via the EGFR/AKT/ERK signaling pathway 17. In esophageal cancer, miR-133a could targeted EGFR, and thus promote cell apoptosis and radio-sensitivity via downregulating the MEK/ERK pathway 5. MiR-133a also served a tumor suppressor by modulating the ERalpha and AhR signaling pathways in breast cancer cell line MCF-7 18. MiR-133a increased apoptosis and inhibited proliferation in colorectal cancer by regulating the RFFL expression, inducing a G0/G1-phase arrest and activating p53/p21 signaling 19. In human osteosarcoma, miR-133a suppressed cell proliferation and promoted cell apoptosis by repressing Bcl-xL and Mcl-1 mRNA 20. MiR-133a-3p was sponged by LINC01278 and the decreased miR-133a-3p could lead to upregulation of parathyroid hormone type 1 receptor (PTHR1), consequently promoting cell proliferation and restraining apoptosis of osteosarcoma cells 4. MiR-133a could suppress cell proliferation in non-small cell lung cancer by binding to the 3'-UTR of YES1 mRNA. YES1, a member of Src family tyrosinekinases (SFKs), could play a prominent role in tumor growth 7. Fused in Sarcoma (FUS) could function as an AR-interacting protein that enhances AR transcriptional activity in prostate cancer. MiR-133a-5p inhibited cell proliferation in AR-positive prostate cancer cell line VCaP and LNCaP by targeting both FUS and AR, which further decreased the resistance to androgen ablation therapies^21^. In pancreatic cancer (PC) cell lines, FEZF1-AS1 served as an oncogene to induce cell proliferation and invasion through miR-133a/EGFR axis under normoxic condition 22. In colorectal cancer, miR-133a-3p inhibited SENP1 expression, and then upregulated CDK inhibitors such as p16, p19, p21, and p27, resulting in the reduction in cell proliferation 6. In GC and PC, ubiquitin-specific protease 39 (USP39), which was negatively regulated by miR-133a, was verified to induce cell proliferation and suppress cell apoptosis through modulating the AKT signaling pathway 10, 23. MiR-133a inhibits the proliferation of gastric cancer cell SNU-1 and promotes SNU-1 cell apoptosis by modulating downstream ERBB2 (also called HER2, a member of EGFR family), and reducing the p-ERK1/2 and p-AKT expression 24.

## MiR-133a in autophagy and metabolism

Autophagy promotes tumor development in multiple cancers via consuming intracellular particles and providing sufficient energy for aggressive behaviors. MicroRNA-133a-3p could target GABARAPL1 to block autophagy-mediated glutaminolysis, further repressing gastric cancer growth and metastasis 25. Forkhead protein 3 (FOXP3), a forkhead transcription factor and specific marker for T regulatory (Treg) cells, can downregulate TP53 by directly targeting its promoter and inhibiting its transcription. In contrast to previous studies, miR-133a-3p could promote the proliferation and autophagy in different gastric cancer cell lines by binding to the 3′-UTR of FOXP3 26. In dedifferentiated liposarcoma (DDLPS), loss of miR-133a could reduce oxidative metabolism, supporting the tumor Warburg effect. Braggio, D. first found that overexpression of miR-133a could increase glycolysis and OXPHOS both *in vitro* and *in vivo*; however, the metabolic change was not sufficient to suppress DDLPS cell proliferation and progression 27. It was known that glycogen metabolism contributed to cancer development. The upregulation of glycogen phosphorylase B (PYGB) was detected in ovarian cancer tissues, while PYGB knockdown suppressed ovarian cancer cells proliferation, invasion and migration. The same procedure was also observed when miR-133a-3p was overexpressed, suggesting its role as a negative regulator in PYGB expression 28.

## MiR-133a in cell migration and invasion

The increased migration and invasion ability accounts for the high mortality rate of cancers. In both *in vitro* and *in vivo* experiments, silencing miR-133a-3p by DNA hypermethylation in breast cancer cell lines and tissues led to increased cell migration, invasion and proliferation. MiR-133a-3p could target and downregulate the mastermind-like transcriptional coactivator 1 (MAML1) mRNA, and then up-regulated DNA methyltransferase 3A (DNMT3A), promoting hypermethylation of the miR-133a-3p promoter 29. The long non-coding RNA X inactive specific transcript (XIST) could function as an oncogene, and promote tumor progression in bladder and pancreatic cancer via regulating miR-133a/EGFR axis 30, 31. MiR-133a-3p inhibited cell invasion and migration in esophageal squamous cancer (ESCC) and oral squamous cell carcinoma (OSCC) via negatively regulating COL1A1, an important factor in extracellular matrix 32, 33. It was hypothesized that epithelial mesenchymal transition (EMT) was vital to cancer migration, with evidence of upregulating expression of E-cadherin, downregulating N-cadherin, vimentin and Slug. Wang, T. et al found higher circP4HB expression in NSCLC tumor tissues than in normal ones, and the role of circP4HB in promoting EMT depended on the suppression of miR-133a-5p 34. Also in GC line SGC-7901 and BGC-823, miR-133a played an antigrowth and antimetastasis role by inhibiting transforming growth factor-beta1 (TGF-beta1)-induced EMT via targeting PSEN1. Moreover, the decrease of PSEN1 could further downregulate Notch 1, Notch 2, and Notch 3 35. MiR-133a could not only suppress cell proliferation, induce cell cycle arrest at G0/G1 stage and accelerate cell apoptosis, but also inhibit cell migration and invasion *in vivo* and *in vitro* via targeting IGF-1R and negatively regulating downstream AKT and ERK signal pathway 36-38. In PC, AFAP1-AS1 was reported to induce IGF1R transcription and activate the AKT/ERK pathways to promote EMT and cell metasis by sponging miR-133a 39. Downregulation of miR-133a-3p functioned as a supportive factor in bone metastasis of prostate cancer (PCa) possibly by targeting EGFR, FGFR1, IGF1R and MET, and inactivating PI3K/AKT signaling pathway 40. MiR-133a could suppress hepatocellular carcinoma (HCC) by targeting Fos-related antigen 2 (FOSL2) and inactivating TGF-β/Smad3 signaling pathway. FOSL2, a member of the AP-1 family, included rich isoforms of Fos and Jun 41. LncRNA HOXD-AS1 could induce NSCLC cell proliferation and migration by targeting miR-133b/MMP-9 axis and promote epithelial ovarian cancer (EOC) cell invasion and EMT by regulating miR-133a-3p/Wnt/β-catenin pathway 42, 43. MiR-133a could suppress breast cancer via targeting LASP1, a actin-binding protein reacting to cAMP and cGMP signaling 44. In ovarian cancer, miR-133a influenced cancer progression and prognosis as a downstream target of plasmacytoma variant translocation 1 (PVT1) 45. The ABHD11-AS1/miR-133a/SOX4 axis played a significant role in the progression in CRC 46. MiR-133a exerted inhibitory effects on gallbladder carcinoma (GBC) via negative regulation of RBPJ, an important transcriptional regulator in the Notch signaling pathway 47. The same modulation found in osteosarcoma contributed to DCs maturation and activation 48. Astragalus polysaccharides (APS) could not only repress osteosarcoma cells proliferation and invasion but also induce cell apoptosis by inducing miR-133a and further inactivating JNK pathway 49. In cervical cancer, miR-133a was also significantlly downregulated. Upregulating miR-133a can restrain the progression of cervical cancer via targeting EGFR and SOX4. NEAT1, a oncogene in various cancers, was reported to sponge to miR-133a and inhibit miR-133a expression, further regulate the cervial cancer progression via the NEAT1/miR-133a/SOX4 axis^50^. Matrine was found to suppress the invasion and metastasis of NCI-H1299 cells by upregulating miR-133a, which further repressed the EGFR/Akt/MMP-9 signal pathway 51. MiR-133a could act as a tumor-suppressor by targeting and downregulating eukaryotic translation initiation factor 4A1 (eIF4A1) 52, matrix metallopeptidase 9 (MMP9) 53, 54, MMP14 55, membrane-type 1 matrix metalloproteinase (MT1-MMP) 56, Fascin1 (FSCN1) 57-59, phosphodiesterase 7A (PDE7A) 60, Coronin-1C (CORO1C) 61 and Sox4 62.

## MiR-133a in drugs and drug sensitivity

On the basis of dysregulation of miR-133a and its tumorsupressive role in various cancers, miR-133a was also a pivotal regulator in drug resistance. Combining drugs targeting miR-133a and traditional chemotherapy may enhance the efficacy of antitumor therapy. MiR-133a could enhance the sensitivity to cisplatin via decreasing the ATP7B expression in Hep-2v cells 63. Upregulating miR133a could reverse the resistance to doxorubicin via decreasing the expression of mitochondria uncoupling protein 2 (UCP-2) in doxorubicin-resistant breast cancer cell line MCF-7/Dox 64. MiR-133a overexpression could enhance the sensitivity to ADM in HepG2 cells by inhibiting multidrug resistance-associated protein 1 (ABCC1), a ATP-binding cassette (ABC) transporter of cellular drug disposition 65. TNF-related apoptosis-inducing ligand (TRAIL), a new anti-cancer drug, could specifically induce cancer cell apoptosis. MiR-133a was found to promote TRAIL resistance in glioblastoma via down-regulating DR5 expression and activating NF-κB signaling 66.

## MiR-133a in cancer prognosis

Accumulating evidence has suggested the association between miR-133a and poor overall survival (OS) of patients with solid cancer 67, 68. A meta-analysis revealed that high expression of miR-133a was related to better prognosis and ameliorated clinicopathological features in digestive system cancers^69^. A research in 110 patients showed that the reduced serum miR-133a contributed to poor prognosis of pancreatic cancer (PC), indicating that miR-133a might function as a specific diagnostic indicator for PC 70. The terminal differentiation induced ncRNA (TINCR), a target of miR-133a, was associated with shorter disease-free survival (DFS) and OS of HCC patients 71. MiR-133a was also found to be associated with the prognosis of bladder cancer, as well as better survival and less resistance to treatment in prostate cancer 72, 73. The expression of miR-133a was negatively correlated with lymphatic metastasis, clinical stage and MMP-14, EGFR levels of NSCLC 74, 75.

## MiR-133a as a potential biomarker

Plenty of efforts have been devoted to evaluating the efficacy of miR-133a as a diagnostic marker. By conducting a case-control investigation in 50 pairs of breast cancer and normal tissues, Bitaraf, A. et al. considered miR-133a-3p, along with miR-127-3p, miR-155-5p, miR-199b-5p, and miR-342-5p, were promising biomarkers for BC 76. MiR-133a-3p was significantly modulated in triple-negative breast cancer (TNBC), suggesting that miR-133a-3p might be a predictor of cancer invasion and prognosis 77. Early stage renal cell carcinoma is often failed to be diagnosed due to its asymptomatic feature. Luckily, miR-133a-2 was a newly found biomarker of renal cell carcinoma (RCC) 78. At present, it is still hard to estimate the metastatic potential of gastrointestinal neuroendocrine neoplasms (GI-NENs), yet a lower level of miR-133a was detected in 51 primary GI-NENs with liver metastases, and a higher level of miR-133a was found in appendiceal carcinoids without metastases compared to the primary tumors 79. A multicenter, retrospective research proposed that circulating microRNAs like miR-133a were more sensitive in diagnosing preclinical and early-stage hepatocellular carcinoma than AFP20 80. Interestingly, the methylation-silencing of miR-133a could be reversed by H. pylori eradication, suggesting the potential role of miR-133a in H. pylori-induced gastric carcinogenesis 81. Combined with gastroscopy, miR-133a in gastric juice could also be a novel test target for screening gastric cancer 15, 82. The combination of miR-133a and FOBT could be a potential detection mode in colorectal cancer screening 83. Based on the information from The Cancer Genome Atlas (TCGA) database and Meta-analysis results, along with bioinformatics analysis, miR-133a was recognized as a potential biomarker of NSCLC 84-86, oral cancer 87, 88, osteosarcoma 89, cervical cancer 90, prostate cancer 91, digestive tumors 92 including EC 93-95, GC 96, 97, pancreatic ductal adenocarcinoma (PDAC) 98 and CRC 99. A study of 8006 tumors including 19 tumor types suggested that hypoxia contributed to genomic instability. Among the dysregulated hypoxia-associated microRNAs, miR-133a-3 was considered to be a novel prognosis biomarker for hypoxic tumors with elevated risk for distant metastasis 100. MiR-133a-3p dysregulation was also found in smoking-induced HPV (+) oropharyngeal cancer patients^101^. Taken together, the dysregulation of miR-133a occurs at the early stage of tumorigensis, and low expression of miR-133a frequently indicates poor prognosis, which is consistent with its tumorsuppressive role in cancer development and progression. MiR-133a might serve as a promising biomarker for cancer early diagnosis, monitoring cancer progression and treatment response.

## MiR-133a mediating different signaling pathways

Studies focusing on the potential mechanism of miR-133a described EGFR as a promising target gene and the downstream signaling pathway. P53 was predicted to bind to the promoter of miR-133a-1, a precursor of miR-133a-3p, and thus activate miR-133a-3p expression. C-Myc, an oncogene, could influence p53 transcription, suppress P53 expression, and further downregulate miR-133a-3p expression. MiR-133a-3p could directly target the mRNA of EGFR 16, 102, inactivate the EGFR/RAS/ERK/c-Myc pathway or EGFR/PI3K/AKT/c-Myc pathway and increase p53 expression. VPS33B, a member of Sec-1 domain family, could activate miR-133a-3p by inducing p53 nuclear translocation, further regulate the EGFR/RAS/ERK/c-Myc/p53/miR-133a-3p feedback loop, and thus suppress CRC progression 103. VPS 33B could also inhibit cell proliferation and chemoresistance to fluorouracil (5-FU) both *in vivo* and *in vitro* by regulating EGFR/PI3K/AKT/c-Myc/P53/miR-133a-3p signaling loop 104. In nasopharyngeal carcinoma (NPC), cinobufotalin (CB) induced FOXO1-mediated cisplatin sensitivity by down-regulating MYH9 which was colocalized with FOXO1 in the cytoplasm via the PI3K/AKT/c-Myc/P53/miR-133a-3p pathway 105. Activation of CXCL12/CXCR4 axis upregulated LncRNA XIST, which acted as a ceRNA to sponge miR-133a-3p, and promote malignant progression of CRC cells via lncRNA XIST/ miR-133a-3p/RhoA axis. RhoA, a potential target of miR-133a-3p, could regulate cell motility through cytoskeletal reorganization by promoting actin polymerization and actomyosin contractility via the ROCK/p-MLC pathway 106.

## Conclusions

MiR-133a is significantly downregulated in malignant cancers and often accompanied by poor prognosis. It also participates in various biological processes, including proliferation, apoptosis, autophagy, migration, invasion and drug resistance, which rely on the regulation of downstream target genes and signaling pathways, with the EGFR/c-Myc/P53 axis as the commonly seen one. Accumulating studies have shown that miRNA may serve as an effective molecular targeted drug for the treatment of cancers because the recovery of abnormally expressed miRNA levels can affect the onset, development and metastasis of multiple cancers 107. We hereby suggest that miR-133a might be a potential biomarker and therapeutic target in maligant tumors, and new attempts like combining miR-133a with traditional cancer therapy should be made.

## Figures and Tables

**Figure 1:**
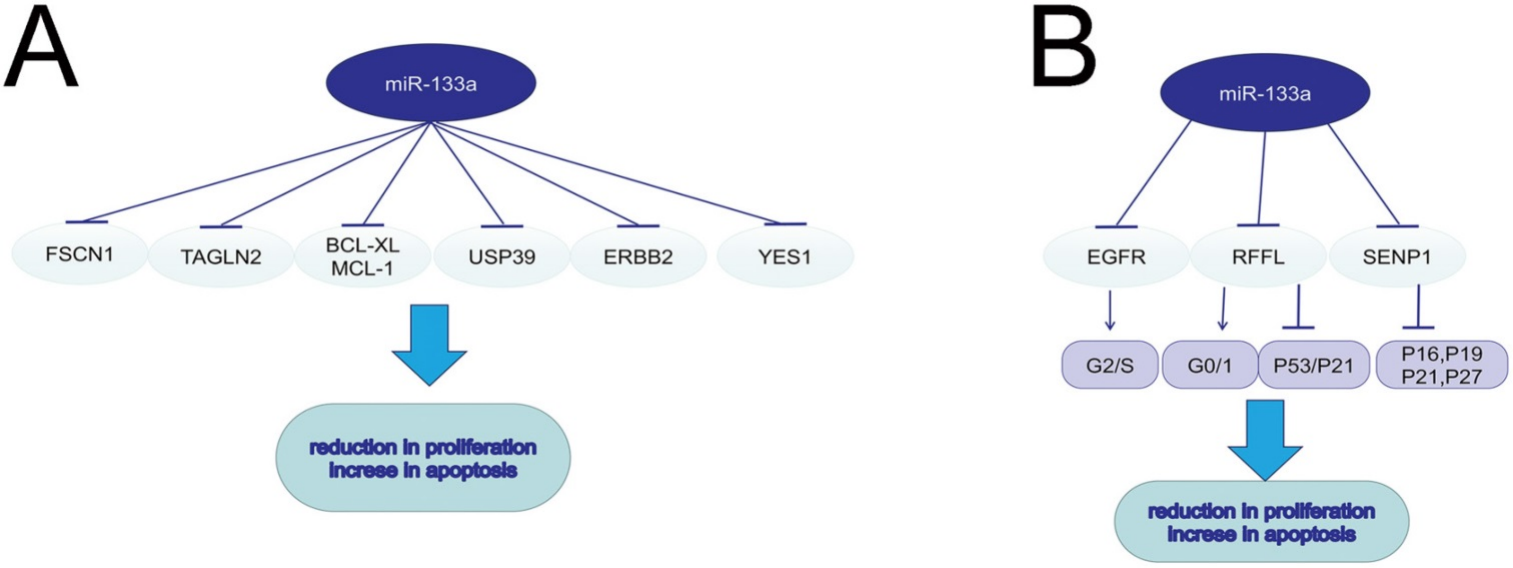
The role of miR-133a in cell circle, proliferation and apoptosis.

**Figure 2:**
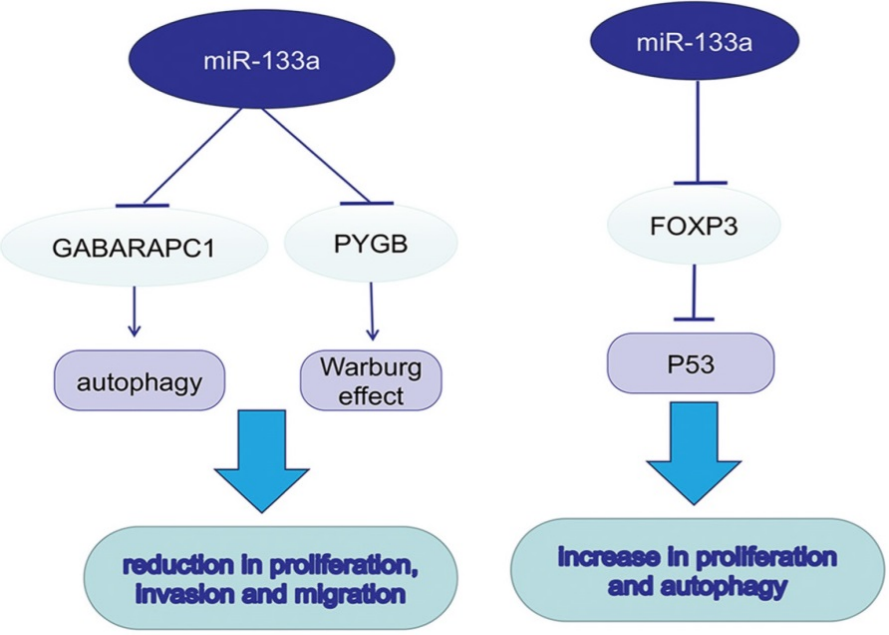
miR-133a promotes autophagy and the Warburg effect, which further promotes tumor progression.

**Figure 3:**
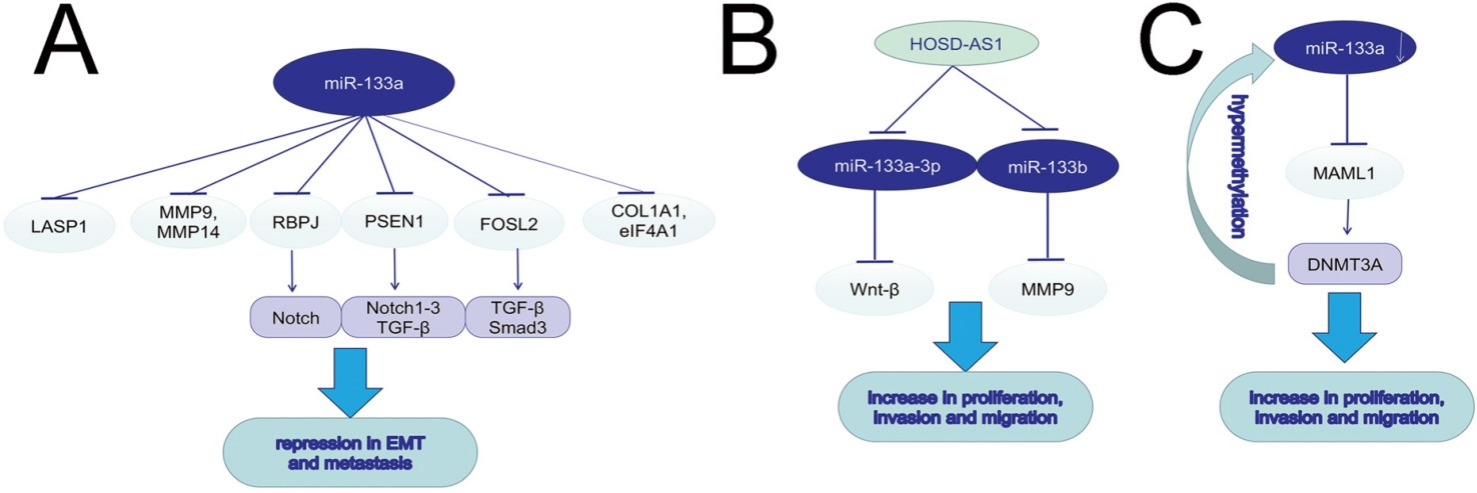
miR-133a induces EMT, invasion and migration in various tumors.

**Figure 4:**
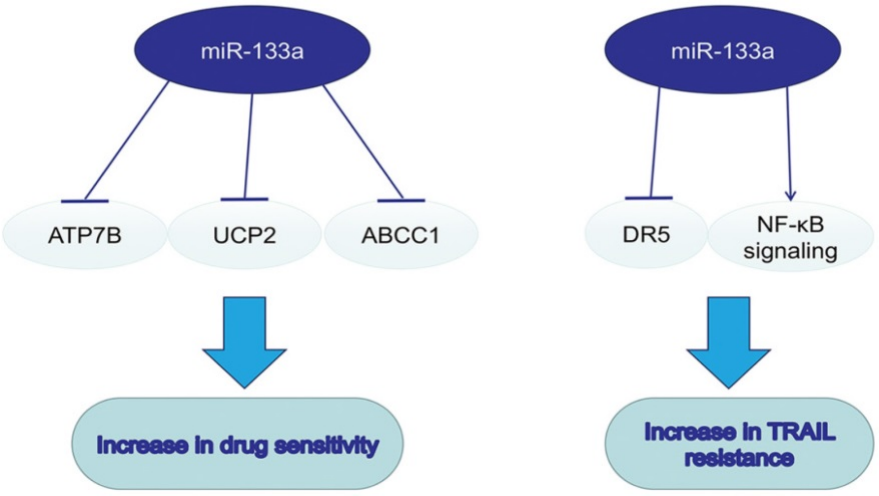
The dual role of miR-133a in drug resistance.

**Figure 5:**
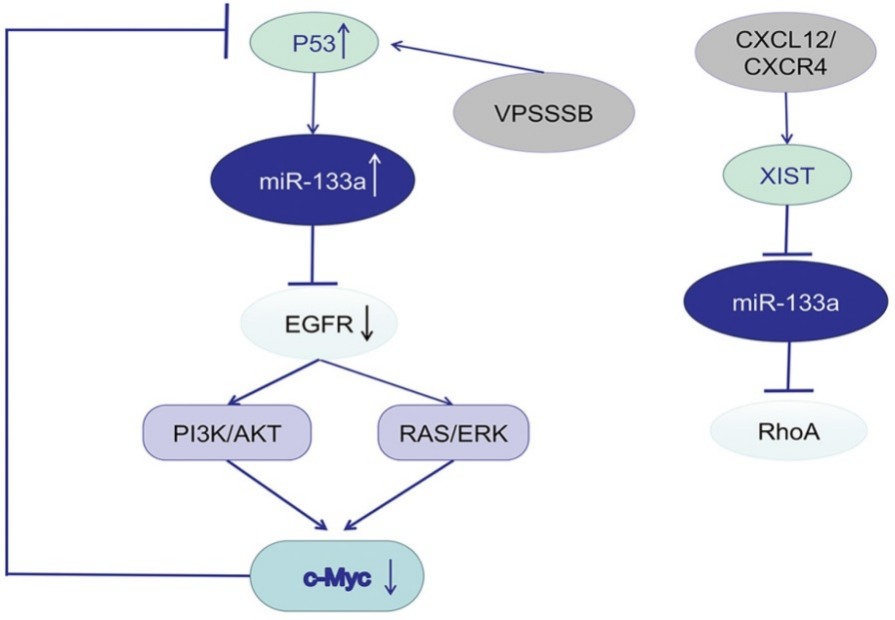
miR-133a involves in EGFR/c-Myc/P53 axis and the associated signaling pathway.

**Table 1:** Target genes of miR-133a in various cancers

MiR-133	Cancers	Target genes
Downregulated	CRC	SOX4, FSCN1, RFFL, SENP1, Eif4A1, Rho A
	Bladder cancer	FSCN1, TAGLN2, EGFR, GSTP1
	GC	FSCN1, USP39, ERBB2/HER2, GABARAPL1, FOXP3, DSEN1, TAGLN2, Sp1
	Nasopharyngeal cancer	EGFR
	ESCC	FSCN1, EGFR, COL1A1, SOX4
	RCC	TAGLN2
	Breast cancer	EGFR, MAML1, LASP1, UCP2
	NSCLC	EGFR, YES1, CORO1C
	Osteosarcoma	BCL-xl, Mcl-1, RBPJ, PTHR1
	PC	EGFR, USP39, IGF1R
	Ovaria cancer	PYGB, PVT1
	OSCC	COL1A1
	Prostate Cancer	EGFR, FGFR, IGF1R, MET
	HCC	FOSL2, MMP9, FSCN1, ATP7B, ABCC1
	GBC	RBPJ
	Glioma	MMP9, MT1-MMP
	Endometrial cancer	PDE7A
	Cervical cancer	EGFR, SOX4

CRC: colorectal cancer; GC: gastric cancer; ESCC: esophageal squamous cancer; RCC: renal cell carcinoma; NSCLC: non-small cell lung cancer; PC: pancreatic cancer; HCC: hepatocellular carcinoma; GBC: gallbladder carcinoma.

**Table 2:** Summarization of lncRNA-miR-133a interactions in human cancers

Types of cancer	LncRNA	Expression	Reference
osteosarcoma	LINC01278	upregulated in tumor tissues and cells	4
PC	FEZF1-AS1	upregulated in tumor tissues and cells	22
Bladder cancer, PC, CRC	XIST	upregulated in tumor tissues and cells	30,31,106
PC	AFAP1-AS1	upregulated in tumor tissues and cells	39
NSCLC, ovarian cancer	HOXD-AS1	upregulated in tumor tissues and cells	42,43
CRC	ABHD11-AS1	upregulated in tumor tissues and cells	46
cervical cancer	NEAT1	upregulated in tumor tissues and cells	50

CRC: colorectal cancer; NSCLC: non-small cell lung cancer; PC: pancreatic cancer.
